# Uncovering How Social Cognitive Representations of Bilingualism in the United States Can Result in Psychological Shame and Linguistic Homelessness for Transnational Youth: Reorienting Bilingualism-as-Problem to a Resource and a Right

**DOI:** 10.3390/bs16050674

**Published:** 2026-04-29

**Authors:** Steve Daniel Przymus, Omar Serna-Gutiérrez, Pablo Montes

**Affiliations:** 1 Feinstein College of Education, The University of Rhode Island, Kingston, RI 02881, USA; 2 College of Education, Texas Christian University, Fort Worth, TX 76129, USA; omar.serna@tcu.edu (O.S.-G.); p.montes@tcu.edu (P.M.)

**Keywords:** bilingualism, metonymy, metaphor, mycelial networks, translanguaging, transnational youth

## Abstract

Language is social, as it is used by individuals to communicate and exchange ideas in society. Language is also cognitive, as the primary function of language, even before communicating and exchanging ideas, is to think. This article connects the social representations of what bilingualism is in the United States and how transnational youth are talked about in U.S. society with how both of these social representations create cognitive representations (e.g., thoughts, ideas, and beliefs) about transnational youth that result in negative educational policies and practices and shameful psychological and behavioral experiences for these youth. We begin with an ethnosemantic analysis of the word “bilingual” in the U.S. and then use the cognitive linguistic phenomena of conceptual metaphor and conceptual metonymy to explain how bilingualism is cognitively viewed as a “shameful problem” in society for transnational youth. We link linguistic shame, brought on by the social cognitive representations of bilingualism as transnational youth metonymically being incomplete, broken, in disrepair, fractured, unsettled, displaced, lacking fully built linguistic structures, not fully in possession of any language, to the phenomenon of and conceptual metaphor of TRANSNATIONAL YOUTH’S BILINGUALISM IS LINGUISTIC HOMELESSNESS. We conclude by putting forth a new metaphor, TRANSNATIONAL YOUTH FUNDS OF KNOWLEDGE ARE MYCELIAL NETWORKS, that rejects the concept of linguistic homelessness by pointing to these youth’s expanding networks of fluid languaging practices, transnational academic skills, and ever adapting identities. Through this new discourse, we advocate for new ways of socially talking about transnational youth and their languaging practices that may lead to different cognitive representations of these students; reorienting bilingualism from a problem to a resource and a right.

“Society is a system for designating allotted places, and humiliation puts everyone in their place and makes them aware of their inferior status.”

(Gros, 2025, p. 22)

## 1. Introduction

### 1.1. Pulling the Contributions of the Special Issue Together

Throughout the articles in this special issue on the language, education, and identity development of transnational youth,^1^ there is a common thread of these youth’s language, and thus identity, being seen and experienced as some kind of problem to be solved. In their contribution, Mora-Pablo et al. (2025) uncover how language and identity are used as gatekeeping mechanisms that reify “Eurocentric, nation-bound assumptions about pedagogy, belonging, and professional development” for preservice teachers (many of whom are former transnational students themselves) who will work with transnational youth (p. 1). In addressing the need for a change in organizational behaviors in the preparation of teachers, the authors call for teacher preparation programs to adopt decolonial practices that “can cultivate culturally responsive pedagogies that transcend national boundaries” (p. 1). Similarly, Catalano et al. (2026) describe a transdisciplinary arts-based approach, using Forum Theater, as a way that teacher educator programs can prepare teachers to meet the needs of transnational students. Each Forum Theater scenario that preservice/in-service teachers practice targets specific negative experiences (e.g., discrimination, racism, stereotyping, etc.) faced by transnational students in schools. In his special issue article, Frausto-Hernandez (2025) also seeks to provide insight into the cognitive, behavioral, academic, and identity challenges that transnational youth experience. Frausto-Hernandez’ “ethnographic self-reconstruction recounts a personal story as a transnational,” often reflecting upon how his own and other transnational students’ languaging practices and identities were used to “other” them with identities of being different and/or of having done something wrong, emphasizing their own feelings of doubt, uncertainty, and emotional scars. Again, like Mora-Pablo et al. (2025), Frausto-Hernandez provides a solution to problems faced by transnational youth by putting forth a framework for the concurrence of the interrelated elements of transnationalism, resulting in the potential “development of *conocimiento*, as well as the [re]construction of the identity of an individual,” resulting in what Frausto-Hernandez calls “transnational capital” (p. 19).

Other contributions to this special issue, such as Palala Martínez and Hamann’s (forthcoming) use of multilingual poetry workshops, digital storytelling, AI, and creation of trilingual educational materials through a Youth Participatory Action Research (YPAR) approach; Jacobo Suárez and Despagne Broxner’s (2026) reporting on how the educational trajectories of transnational youth, who return to México after having formative schooling experiences in the United States, influences these students’ Spanish language maintenance, English acquisition, socialization processes, and identity development; and Solmaz’ (2026) creative and original application of fictional linguistic landscapes (FLL) for finding a way to take aim at difficult issues faced by transnational youth and their language, academic, and identity development—all deeply and meaningfully touch on the theme of somehow solving the challenges and problems that transnational youth’s language (bilingualism) causes in schools and society. And although these contributions are critical to improving transnational youth experience in schools and society, they do not inherently address the social and cognitive roots underlying why these students’ struggles exist in the first place.

In current American society, problems associated with transnational and immigrant individuals are commonly front and center in U.S. media and politics. “Negative discourse on immigrants is affecting how they are viewed in schools and society, with talk of them being murderers, criminals, or animals” (García et al., 2025, p. xiii). It is no wonder that educational researchers and educators have gotten caught up in the “finding the solution to problems” cycle, and although we are grateful for and applaud the “problem solving” work represented in this special issue, we wonder about the implicit cognitive linguistic phenomena underlying the negative discourse that go unquestioned and allow for the normalization of deficit-based ideologies regarding transnational youth.

### 1.2. Why a Social Cognitive Approach Is Needed

In this paper, we apply a social cognitive theory (Bandura, 1986) approach to uncovering and explaining the underlying linguistic mechanisms that provide the basis for the bilingualism-as-problem orientation attributed to transnational youth in U.S. schools. We take this approach because we recognize the overall influence of societal language on societal thought and that “most external influences affect human functioning through intermediary self processes rather than directly” (Bandura, 1999, p. 185). To say it in a different way, how transnational youth and their languaging practices are talked about in society can indirectly (and also directly) influence how these youth see themselves, their identities, their possibilities, and their place in society. However, this is not a “one-sided determination” (Bandura, 1999, p. 185). Transnational students’ bilingualism, behaviors, identities, etc. may be framed as a problem to solve, but any break or change in the narrative (brought on by educators, researchers, or even transnational students, themselves) can change the narrative and resulting reality (Przymus, 2025). “In the case of the imposed environment, certain physical and sociostructural conditions are thrust upon people whether they like it or not. Although they have little control over its presence, they have leeway in how they construe it and react to it” (Bandura, p. 186). We propose, here, both a description of and a break in the normalized and unquestioned rhetoric of transnational youth’s language practices and identities, thus providing them with positive ways of construing and reacting to their new environments. Consistent with work in social semiotics and the tradition of mythologies that drive societal thought (Barthes, 1957/1972) and the potential for “remythification” (Przymus, 2025), we conclude this article with a new narrative, a new discourse, a new myth, and a potential new reality for transnational youth. 

### 1.3. A Structural Overview of This Paper

We have divided this paper into three parts: 1. An ethnosemantic analysis of the word “bilingual,” as a place to start investigating how individuals’ ideologies about what it means to be bilingual start with unquestioned and normalized definitions of words; 2. A cognitive linguistic application of the cognitive linguistic phenomena of conceptual metaphor and conceptual metonymy to explain how bilingualism is cognitively viewed as a “shameful problem” in society to solve for transnational youth, much like “homelessness,” leading to the influential conceptual metaphor of TRANSNATIONAL YOUTH’S BILINGUALISM IS LINGUISTIC HOMELESSNESS (Bakhtin, 1981; Baratta, 2014; Britton, 1996). We link linguistic shame, brought on by the social cognitive representations of bilingualism for transnational youth as a personal problem that results in personal shame, much like how the discourse around being homeless or unhoused is often focused on the individual’s problem or fault for being unhoused, to the phenomenon of “linguistic homelessness;” and 3. We conclude with a remythification of the “linguistic homelessness” myth to a new conceptual metaphor of TRANSNATIONAL YOUTH FUNDS OF KNOWLEDGE ARE MYCELIAL NETWORKS and a new myth of “network of homes” as a new way of socially talking about transnational youth and their languaging practices that may lead to different cognitive representations of these students, reorienting bilingualism from a problem to a resource and a right.

## 2. Part 1: An Ethnosemantic Analysis of the Word “Bilingual” and Its Social Cognitive Influence in the United States

**Figure 1:**
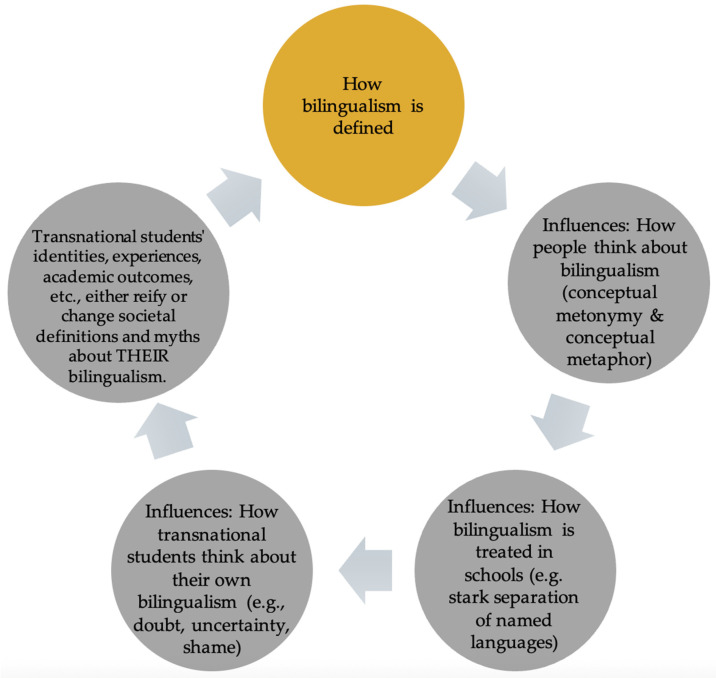
Social cognitive cycle of ideologies of bilingualism as problem, resource, and right.

Ruiz (1984), in his seminal work “Orientations in language planning,” proposes three orientations (language-as-problem, language-as-right, and language-as-resource) that have since been influential guideposts in language planning policy around the world. Ruiz claims that “While problem-solving has been the main activity of language planners…rights-affirmation has gained in importance with the renewed emphasis on the protection of minority groups” and that the third orientation of viewing language-as-resource should receive more attention, especially in the United States (Ruiz, 1984, p. 15). As pointed out in the introduction, over 40 years later, we believe that researchers and educators are still caught up in the language-as-problem orientation and in the cycle of reactively trying to solve or address that problem. We believe that this cycle begins with how bilingualism is defined (see Figure 1). In this first section, we try to identify the root of the *bilingualism-as-problem* orientation theme that runs through many contributions of this special issue on the language, education, and identity development of transnational youth. We start by conducting an ethnosemantic analysis of the word “bilingual.”

### 2.1. The Social Cognitive Meaning of “Bilingual”

Ethnosemanticists “see the linguistic code as a reflection of the cognitive code: by studying how we talk, especially how we name things, ethnosemanticists say they can discover how we think, and ultimately how we see the world” (Ruiz, 2017, p. 221; see also Frake, 2013). Ruiz (2017) continues by claiming that “there is no way to know what people are really doing unless you know what they think, and you find out how they think by how they talk” (p. 223). How society names and defines bilingualism may have an important implicit impact on how society (e.g., educational policy makers, educators, researchers, transnational students, families, etc.) thinks about and acts on bilingualism. Following the ethnosemantic breadcrumbs that make-up the word “bilingual,” we can uncover the reflection of the linguistic code on the cognitive code and start to understand orientations of bilingualism as a problem, a resource, or a right. What follows is an ethnosemantic analysis of the most commonly used dictionary definition of the words “bilingual,” then “bi,” and finally “lingual.” By representing the definitions exactly how they are presented in the dictionary and breaking down the morphemes of bilingual, we can show how a morpho-ethnosemantic deconstruction of *bilingual* helps to understand how American^2^ society has constructed the meanings and ideologies that drive thoughts, practice, and policy that can negatively impact transnational youth. For each definition, we have purposefully *italicized* words and phrases that cognitively influence overall meaning. Merriam-Webster (n.d.a) defines “bilingual” as
**bilingual****adjective**bi·lin·gual (ˌ)bī-ˈliŋ-gwəl 1: having or expressed *in two languages*a bilingual documentan officially bilingual nation


2: *using or able to use two languages especially with equal fluency*bilingual in English and Japanese


3: of or relating to bilingual education
Alone, the influence of this definition on the language socialization and ideology formation of society is quite telling. This definition reifies the time-valued concept that to be bilingual is to be *equally fluent* in *two* named, official languages (italicized, above). This definition also alludes to a sense of “ableism,” or a discrimination, prejudice, and devaluation of anyone who falls outside of this equally fluent, official, able-bodied, able-linguistic, standard, etc., way of being. However, this socialization and cognitive formation of what “bilingual” is goes even deeper. Merriam-Webster (n.d.b) defines “bi” as
**bi-****prefix**1a**:** *two**bi*lateral1b**:** *coming or occurring every two**Bi*centennial1c**:** *into two parts**Bi*sect


2a**:** *twice* **:** *doubly* **:** *on both sides**Bi*convex2b**:** *coming or occurring two times**bi*annualcompare semi-



3**:** *between, involving, or affecting two (specified) symmetrical parts**Bi*labial


4**:** containing *one (specified) constituent in double the proportion of the other constituent* or *in double the ordinary proportion*
Again, with this definition, we italicize words and phrases that act to solidify the idea of bilingualism having to be “*symmetrical*,” “*double the ordinal proportion*,” and having *two sides*; a metaphorical or social/external cognitive perspective of two distinct linguistic domains. We do this to show how and why the social/external perspective continues to dominate cognitive ideologies and practices through unquestioned, normalized words and definitions. Also, the word “constituent” in this definition is used to refer to linguistic structures that are equal in number, size, and quality that belong to the idealized, distinct linguistic domains of a balanced bilingual individual. The foundation for the myth of the perfectly balanced bilingual is the metaphor that bilinguals have built perfectly constructed and symmetrical walls, scaffolding, and structures for their sound houses or linguistic homes. This, then, allows for the metaphor of linguistic homelessness for any individual, such as transnational youth, whose language practices fall outside of this mythical, perfect, balanced, idealized model.

To continue this ethnosemantic analysis of “bilingual,” below, we include the Merriam-Webster (n.d.c) definition of “lingual.”
**lingual****adjective**lin·gual ˈliŋ-gwəl


1a**:** of, *relating to, or resembling the tongue*1b**:** *lying near or next to the tongue*especially**:** relating to or being the surface of tooth next to the tongue1c**:** *produced by the tongue*


2**:** linguistic*lingually* (adverb)
Adding the various definitions of “lingual” fully completes the picture of “bilingual,” and thus a “bilingual person,” is someone who has *two tongues*, that produces *two distinct* “official” languages *symmetrically*, *equally*, on *both sides*, with *two constituents*, and in *double* the “*ordinary*” proportion. This, again, is only a political/social/external, metaphorical cognitive understanding, or half the story, which leaves out and metonymically erases what bilingual individuals do internally when they dynamically language across named languages and across political/linguistic boundaries, or translanguage (García, 2017; Przymus, 2024).

#### The Relationship Between Definitions, Social Cognitive Understandings, and Ideologies Toward Transnational Youth

This impact of these definitions of bilingualism, this example of how we call things affecting how we think about things, has had a tremendous historical impact on some populations of bilinguals around the world.^3^ For those who occupy a socially, racially, ethnically, etc., subaltern status in society, these definitions provide evidence for positioning these individuals as “a-lingual” or not fluent in any language (Garcia-Louis & Mateos-Campos, 2025; Torres, 2018; Zentella, 1995), as “semilingual” or not quite there (and never will fully get there) in any language (Garcia-Louis & Mateos-Campos, 2025; Torres, 2018; Zentella, 1995), as broken or fractured (Bayram et al., 2021; B. B. Flores et al., 2022; Przymus, 2024), as dis/abled (Cioè-Peña, 2021; Soto-Boykin et al., 2023; García et al., 2024), as ni-linguals (McNeil, 2008; Perez Firmat, 1994) or not fully or officially possessing any language, as…a problem. Nilingualism (McNeil, 2008) derives from the Spanish word *ni*, meaning neither, being used as a prefix for lingüe, instead of *bi* for bilingüe, to mean not possessing either of the two languages that one is exposed to. In a similar ethnosemantic analysis, Perez Firmat (1994) describes “Spanish includes a term… Nilingüe. Just as a bilingüe is someone who speaks two languages (say, Spanish and English), a nilingüe is someone who doesn’t speak either: ‘ni español, ni inglés’” (p. 45). Perez Firmat concludes by saying that a nilingüe “is homeless in two languages” (p. 46), a conceptual metaphor that we explain in the following section.

## 3. Part 2: A Cognitive Linguistic Explanation of the TRANSNATIONAL YOUTH’S BILINGUALISM IS LINGUISTIC HOMELESSNESS Metaphor

### 3.1. The Cognitive Linguistic Phenomena Underlying and Creating Ideologies

#### 3.1.1. Conceptual Metaphor

**Figure 2:**
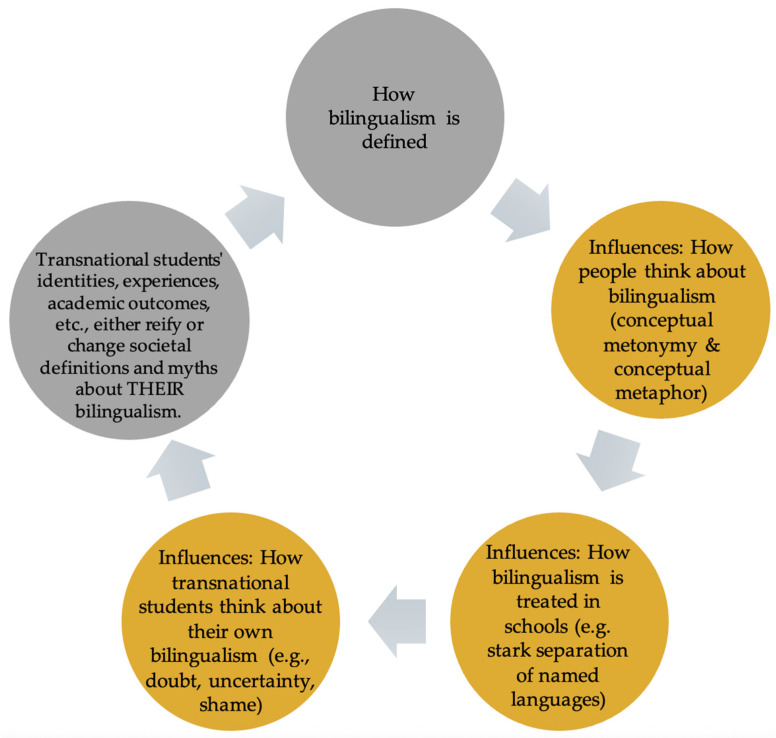
Social cognitive cycle of ideologies of bilingualism as problem, resource, and right.

**Figure 3:**
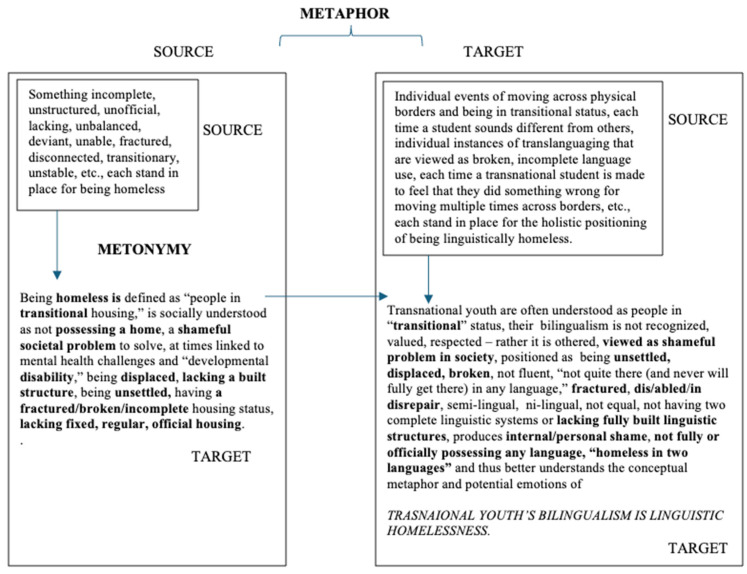
Cognitive relationship between conceptual metonymy and conceptual metaphor in the TRANSNATIONAL YOUTH’S BILINGUALISM IS LINGUISTIC HOMELESSNESS metaphor. Adapted from (Matusz, 2020) “*I will see it done*: Metonymic extensions of the verb *see* in English in (Przymus et al., 2026). Homeless definition (U.S. Department of Housing and Urban Development, 2011).

Arguably, life is complex. Humans make sense of a complex world by relating life processes and experiences through stories made up of conceptual metaphors, that in turn influence our actions and conceptions of self and others (see Figure 2). When individuals want to understand heartbreak, the conceptual metaphor “LOVE IS A BATTLEFIELD” is employed (Benatar, 1983). Likewise, “most people will have heard that life is a highway or journey and understand that one’s life consists of a long road of experiences that has hills, bumps, and curves along the way” (Przymus et al., 2026, p. 9). In both of these examples, the abstract target concepts of “love” and “life” are understood through the more concrete source concepts of “battlefield” and “highway.” Santa Ana (2002) defines metaphor as “a conceptual mapping from a semantic source domain to a different semantic target domain” (p. 26). In Figure 3, we use the metaphor that we are exposing in our paper, TRANSNATIONAL YOUTH’S BILINGUALISM IS LINGUISTIC HOMELESSNESS, to explain how abstract target concepts (e.g., the linguistic homelessness of transnational youth) are understood by mapping them onto concrete source concepts (e.g., the real homelessness experienced by the unhoused) via conceptual metaphor. Adding strength to this metaphor is the fact that both concepts are real social issues, thus the social issue of human homelessness acts as an even stronger concrete mental representation for understanding the social, yet more abstract concept of transnational youth being linguistically homeless.

#### 3.1.2. Conceptual Metonymy

However, this conceptual mapping of two related but distinct cognitive domains can only happen if another cognitive phenomenon or conceptual metonymy takes place first. Laying the foundation of the conceptual metaphor of linguistic homelessness are all of the individual metonymic parts that constitute what it means to be physically homeless (e.g., being in transition, incomplete housing, lacking housing, disconnected, etc., see small box within larger box on the left) and linguistically homeless (e.g., transitional status, translanguaging viewed as broken, incomplete language use, being made to feel shame for having done something wrong, etc., see small box within larger box on the right). Metonymy is “when one thing stands for another thing to which it is related or closely associated” and it also functions as a means of “highlighting or backgrounding certain aspects of an event, action, or person” (Catalano, 2017, p. 127). This “stands for” function of metonymy, known as synecdoche, creates fast mental shortcuts for understanding a whole concept through just seeing or hearing physical or linguistic parts of the concept.

Metonymy needs to be first considered and understood, whenever social phenomena are explained via metaphors, as metonymy is the basis for metaphors and cognitively happens first, in order for the metaphor to exist (Mittelberg & Waugh, 2008; see also Przymus et al., 2026). Metonymy is a cognitive process that creates “natural inference schemas,” which act as the basis for implicit meaning making (Panther & Thornburg, 2005, p. 353). Whereas the cognitive process of conceptual metaphor is to map or link two distinct cognitive domains (i.e., source and target domains; see two separate large boxes in Figure 3), conceptual metonymy “maps or relates two concepts that belong to the same cognitive domain and thus needs to happen first in order for the distinct cognitive domains of metaphor to exist (Barcelona, 2000; Przymus & Huddleston, 2021)” (Przymus et al., 2026, p. 3). This is visually illustrated in Figure 3 by showing that within the one, separate cognitive domain “homeless” (large box on left), which serves as the source domain for the overall metaphor, there are also intra-domain source concepts (small box) that stand in place for the overall target concept of “homeless,” which then allows the metaphor to pick up this concept as the concrete source for linking and understanding the abstract target concept of “linguistic homelessness” (large box on the right). This metonymic process of within-domain or intra-domain mapping not only creates quick mental shortcuts for understanding the cognitive concept of “homeless” on the left, but it also happens within the cognitive domain of “linguistic homelessness, on the right. Inside of the cognitive domain of “linguistic homelessness” (large box on the right in Figure 3), we can also see that the metonymic process of intra-domain mapping is taking place. Simply by stating part of the overall concept (e.g., a teacher stating that a transnational youth’s translanguaging is evidence of broken speech), the entire concept of “linguistic homelessness” is ignited and brought to the forefront of thought. Through the cognitive mapping or linking, first through metonymy, then through metaphor, of the concrete cognitive source concept of homelessness to the abstract, cognitive target concept of linguistic homelessness, we now can understand how this could be applied to transnational youth’s bilingualism, simply through the use of any individual words or phrases in discourse, such as “fractured,” “in disrepair,” “unsettled,” “broken,” “transitional,” “lacking fully built linguistic structures,” “*not in possession* of an official language,” resulting in a societal paradigm and ideology of these youth and their bilingualism being some kind of a problem (like societal homelessness) to solve.

This overall metaphor for transnational youth’s bilingualism (and by default identity) as a problem to solve does not only affect how teachers and educational policy makers feel about them, it also impacts how these youth feel about themselves. Santa Ana (2002) points out that “[c]rucially for language-minority populations and for all concerned citizens, metaphor is central to the way people talk about themselves and their social world. As such it is the key element with which discourse constructs the social world” (p. 43). In the next section, we foreground how the above metonymies and metaphors create psychological shame within transnational youth. We then end our paper with how, using a new way of talking about these youth’s bilingualism and funds of knowledge, through the creation of new metonymies and a new metaphor, this shame of linguistic homelessness can be converted to a pride and strength that comes from the recognition of multiple diverse linguistic, cultural, and identity networks and homes.

### 3.2. An Empire of Shame

#### 3.2.1. Raciolinguistic Ideologies and Zozobra

“I think I understand now…that the bullshit inside of us is nothing but a reflection of the bullshit outside. Or maybe it’s the other way around. In either case, the outside bullshit eventually seeps inside, and settles into the depths of our souls.”

(Naji, 2017, *Using Life*, in Zadie Smith *Dead and Alive*, 2025, p. 102)

What connects most transnational youth to each other, and we will reorient this as a strength of networks below, is that most have lived and even attended school on both sides of the U.S./México border. For many, this subjects them to deficit-based discourses, on both sides, about how they are culturally, academically, and linguistically different. Serna-Gutiérrez (forthcoming) conducts diachronic (throughout time) and synchronic (modern day) discourse analyses of how transnational youth are talked about and traces how raciolinguistic ideologies (N. Flores & Rosa, 2015) on both sides of the U.S./México border construct social identities of these youth as “othered” individuals who experience loneliness and psychic rupture. Pointing to the work of authors, such as Octavio Paz and José Vasconcelos, whose writings are part of the Mexican Secretary of Education required curriculum, Serna-Gutiérrez uncovers how the Mexican mestizo, in general, carries an identity of a colonized subject in Paz’ (1950) *Laberinto de la Soledad*, where Paz describes the Mexican being as grounded in racialized metaphors of submission, abandonment, and shame. Transnational youth, due to their having left Mexico for “el Norte,” upon return to Mexico receive an even worse identity positioning, as a traitor, like the enslaved Indigenous interpreter for Hernán Cortés, La Malinche, herself.

Anchored within colonial logic, on both sides of the border, these youth can often fall outside of the narrow margins of normality. “Normality is not a statistical pattern or a simple average, but a model of respectable behaviour” (Gros, 2025, p. 13). Our world is built on a series of accepted pathological classifications of normal and has been for centuries, if not since humans first started interacting together. Gros, in his seminal work, “A Philosophy of Shame,” often points to how language is paramount in separating those with honor, those who are considered normal, those who society accepts from those who are othered, different, and pushed to the margins of society. “The way you talk…your vocabulary, pronunciation and syntax; the way your mouth moves…will all betray you” and position you as “‘the little people, the lowly, the nobodies’” (Gros, 2025, p. 20; Rostand, 1990^4^). “This marginalization of racialized language practices connects to broader colonial histories that have questioned the linguistic competence of racialized communities as part of their dehumanization” (García et al., 2021, p. 210; Rosa, 2016).

And as if the societal message was not strong enough, schooling policies, procedures, and practices (on both sides of the U.S./México border) often reify these messages of transnational youth and their bilingualism as being broken, incomplete, and a problem to solve. This is accomplished through Standard Spanish ideologies and practices in México, and similarly in the U.S., “the erasure of the home language through English-only school practices reinforces the deficit view that families and their children need to be linguistically ‘fixed’ or ‘repaired’ before they can succeed academically in the United States” (García et al., 2025, p. 133). Again, we see how the language of “fixing” and “repairing” serves the linguistic homelessness metaphor, described above. The shame that transnational youth feel is no mistake, no accident, but rather the result of a powerful tool of an empire that is built on protecting the social, linguistic, and political status quo and keeping people in their place.

The empire has many weapons in its social cognitive arsenal, such as sociopolitical discourses (e.g., metaphors of citizen vs. noncitizen), socioeconomic discourses (e.g., metaphors of wealth vs. poverty), and sociolinguistic discourses (e.g., metaphors of standard vs. nonstandard language), all meant to produce shame about and within the vanquished and colonized. As an example for relating the socioeconomic and sociolinguistic, Gros (2025) states that “It is, above all, the dogmatic neoliberal insistence on individual responsibility and self-reliance that has transformed poverty into a personal defeat, implying shortcomings of character (such as laziness and a lack of self-discipline and moral fiber) that somehow need addressing” (pp. 26–27). Similarly, education in the U.S. is structured to make those who are different, largely and immediately identified via race and language, feel bad about themselves, as if it were something they did or a lack of skills that they possess, perpetually making them feel different and less than, turning “other peoples’ contempt for me into self-contempt” (Gros, 2025, p. 28). This is the TRANSNATIONAL YOUTH’S BILINGUALISM IS LINGUISTIC HOMELESSNESS metaphor that has potential to produce shame every time a transnational student opens their mouth.

In writing about the internalized shame that heritage language speakers (HSs) feel about their languaging practices, often, again, framed as in need of repair, Bayram et al. (2021) claims “it is a shame that HSs should associate words like ‘broken’ with the knowledge they have of their native language, when what they really mean to convey is awareness of the differences they display juxtaposed against a comparison (most likely the illusive monolingual) to which they are not really comparable in the relevant ways” (p. 10). For many transnational youth, this manifests both in the classroom, where their languaging practices are policed, pathologized, or rendered invisible and often when they visit and are compared to monolingual Spanish-speaking family and friends, and are judged as “no sabo kids”^5^ for their different sounding Spanish (Ayala-Saracay, 2025). This experience of shame can lead to what Sánchez (2023, p. 69) calls “Zozobra.”

Zozobra names the feeling of doom, desperation, or uncertainty that defines modern life…in zozobra we are delivered over to a version of the world (which is our version of the world) in crisis—a *divided*, *unsettled* world. This world appears at once as a familiar and unfamiliar, one that we easily recognize but which nonetheless feels uncanny and strangely weird. We find ourselves certain of two things simultaneously: that this is our world *[home]* and that this is not our world *[home]*…Moreover, in zozobra we are overcome not only by uncertainty, *unsettledness*, and disquiet but also by an engulfing sense of sentimentality, a kind of all-consuming mourning for a world that suddenly appears lost and unrecoverable. In zozobra, existing truly feels strange and uncomfortable.

(p. 69, italics, ours)

Again, in the italicized words (*divided, home, unsettled, unsettledness*) we can see how the metaphor of linguistic homelessness is reified in society and ingrained in the bodies and minds of transnational youth, themselves. Zozobra emerges when one is forced to choose between so-called “correct” English and familial Spanish or Indigenous tongues, when neither option fully affirms one’s sense of self.

#### 3.2.2. A Raciolinguistic Perspective and Shift Toward New Discursive Formations

In an attempt to uncover bilingualism-as-a-problem to solve orientation that drives the majority of research on and the teaching of transnational youth, above, we have zeroed in on how bilingualism is defined in the U.S. and have highlighted diachronic (historical) and synchronic (modern day) discourse about transnational youth’s bilingualism that has acted, via the TRANSNATIONAL YOUTH’S BILINGUALISM IS LINGUISTIC HOMELESSNESS metaphor, to produce much shame within these youth. However, just as we have framed this process via a lens of raciolinguistic ideologies, scholars have begun to use these same raciolinguistic ideologies as the foundation for counter narratives, calling for a raciolinguistic perspective. A raciolinguistic perspective

connects a genealogical stance and a materialist framing of race with a focus on language ideologies in order to examine the discursive construction of raciolinguistic ideologies that have historically and continue to co-naturalize language and race in ways that position racialized populations as outside of what it means to be fully human.

(N. Flores, 2022, p. 18)

N. Flores (2022) claims that in adopting a raciolinguistic perspective, one can “denaturalize […] raciolinguistic ideologies in the hopes of developing spaces of resistance that produce new discursive formations that can shape a new grid of intelligibility” (p. 19).

Theoretical and pedagogical spaces of resistance might be found in ways that bilingualism has begun to be talked about, that value holistic languaging practices, such as translanguaging. The act of translanguaging *transcends language as simple linguistic stable structures*, only in spoken and written modes, and only via structured, separated use of societal named languages (e.g., English, Spanish, etc.). In focusing on how speakers DO language and make meaning for themselves with all of their language features (regardless of what named language these are attributed to), translanguaging “unmasks how a traditional language ideology works to exclude/stigmatize/racialize those whose language practices transcend the normed boundaries that have been based on white, mostly male, monolingual speakers considered able” communicators (O. García, personal communication, 1 April 2026). Within this definition of translanguaging, we see language that directly addresses the “linguistic homelessness” framing of transnational youth’s bilingualism by transcending and rejecting ideas of simple *linguistic stable structures,* rather translanguaging focuses on how transnational youth *make/construct* their own language structures (homes) and linguistic networks.

As a means to disrupt the empire of shame, translanguaging is a decolonial process that delinks transnational youth’s bilingualism from the colonial/empire matrix of power (Mignolo, 2013) that uses language to racialize and protect privilege for the powerful. It is within this framing that a new way of talking about transnational youth’s bilingualism, a new metaphor for their ways of being, may emerge and spread.

## 4. Part 3: A Reorienting of the TRANSNATIONAL YOUTH’S BILINGUALISM IS LINGUISTIC HOMELESSNESS Metaphor

“[U]ntil I can take pride in my language, I cannot take pride in myself. Until I can accept as legitimate Chicano Spanish English, Tex-Mex and all the other languages I speak, I cannot accept the legitimacy of myself. Until I am free to write bilingually And to switch codes without having to translate, … my tongue will be illegitimate.”

Anzaldúa (1987, p. 59)

**Figure 4:**
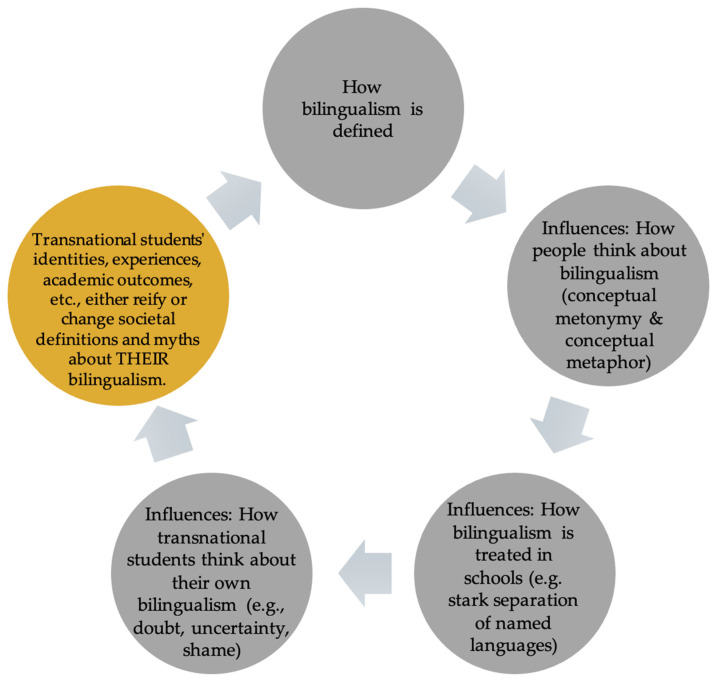
Social cognitive cycle of ideologies of bilingualism as problem, resource, and right.

To this point, we have built an argument that how bilingualism is defined in society influences how people cognitively think about bilingualism, which influences how bilingualism (and by default bilingual transnational youth) is treated in schools, which, in turn, influences how transnational youth think about their own bilingualism, often in terms of doubt, uncertainty, and shame. With this third, and final, section of this paper, we claim that a new metaphor is needed for telling the story about transnational youth’s experiences, languaging practices, and funds of knowledge and that a new social cognitive way of thinking about and talking about transnational youth may change societal definitions and myths about their bilingualism, converting shame into pride and recognized strengths (see Figure 4).

### 4.1. Bilingualism as a Resource and a Right

The benefits and advantages of bilingualism on cognitive abilities (e.g., academic achievement, problem solving, working memory, attention, cognitive flexibility, processing speed, reasoning, etc.) have been well documented (Antoniou, 2019; Bialystok, 2011; Bialystok & Viswanathan, 2009; Chibaka, 2018; Golash-Boza, 2005; Sami & Ahmed, 2025). This research, however, has not always sufficiently reached the classroom where transnational students continue to be compared to either the monolingual, English-speaking, white peer or the emergent bilingual white peer in dual-language and/or foreign language settings and thus are still positioned with identities of not being good enough or lacking in the same linguistic skills that they actually excel in. In fact, the gap between who is able to be bilingual in American society may be growing even wider with the popularity of two-way dual-language bilingual programs, where white, first language English-speaking youth are often celebrated for their emergent bilingualism, while their active bilingual learners of English (ABLE) student peers are held to a different, not-yet (maybe never) proficiency status in standard English identities at school (Przymus et al., 2020). Some researchers in bilingual education have started calling this phenomenon the “gentrification of dual-language education” (Valdez et al., 2016, p. 601). Pointing to the same double standard of who is able to to societally reap the benefits and advantages of bilingualism in the U.S., Golash-Boza (2005, p. 750) points to

the disparate prestige value of bilingual ability. While bilingual ability provides an Anglo-American with valuable cultural capital, the scenario is distinct for Latino and Asian Americans. The meaning given to the use of another language depends not only on the language one switches to, but the person who is doing so.

In order to shift both the social and educational narrative of bilingualism for transnational youth as a problem to that of a resource and a right, more studies are needed that specifically highlight the cognitive advantages of bilingualism for transnational students at school. Examples of such studies to build from include Golash-Boza’s (2005) study that uses data from “the 1992–1993 and 1995–1996 Children of Immigrants Longitudinal Study” (p. 721) to show clear academic advantages for bilingual youth (p. 721), especially in communities of strong bilingual social capital, such as Latinos in Miami, Florida. More recently, Sami and Ahmed (2025) conducted a cross-sectional analysis of 100 five- to seven-year-old children, measuring and comparing the academic outcomes of a monolingual and bilingual group. Based on language development measures and results of the Stanford–Binet Intelligence Scales, they found that bilingual children have “better language development…and a significant cognitive advantage, particularly in areas such as working memory and reasoning” (p. 1).

#### 4.1.1. A New Metaphor

**Figure 5:**
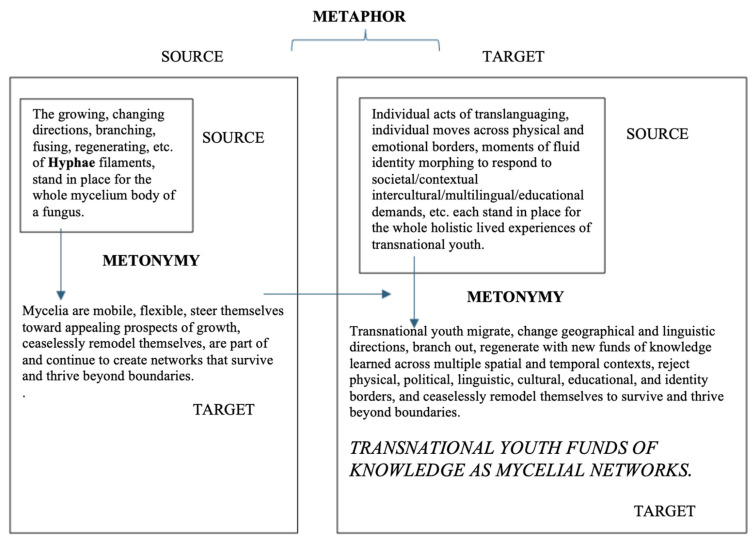
Relationship between conceptual metaphor and underlying conceptual metonymy for the TRANSNATIONAL YOUTH FUNDS OF KNOWLEDGE ARE MYCELIAL NETWORKS conceptual metaphor.

Positioned within a bilingualism as resource and bilingualism as right orientation, we propose a new metaphor, TRANSNATIONAL YOUTH FUNDS OF KNOWLEDGE ARE MYCELIAL NETWORKS, that uses the vast branching and resilient networks of mycelium to visually and mentally represent the vast network of resources that transnational youth have grown across diverse lived experiences. This new metaphor offers a necessary reorientation of how transnational youth’s languaging practices are understood, valued, and enacted in educational spaces. Rather than locating bilingualism within deficit-based narratives that cast transnational youth as linguistically fragmented or incomplete, this metaphor reframes their linguistic and epistemic repertoires as expansive, adaptive, and deeply interconnected systems of knowledge. In doing so, it builds directly on the remythification project outlined earlier in this paper (and expanded upon below), where the shift from “linguistic homelessness” to “network of homes” interrupts the metonymic chain that produces deficit-oriented ideologies. As illustrated above in Figure 3 and below in Figure 5, conceptual metaphors are not isolated linguistic devices. They are rooted in underlying conceptual metonymies that shape how individuals and societies make meaning. When bilingualism is metonymically associated with a lack of something, such as being “incomplete” or “not fully in possession” of a language, it becomes cognitively and socially reified as a problem. The metaphor of mycelial networks reorganizes these cognitive associations but reorients them in a way that cognitively represents strengths and networks of connections, rather than a lack of structures. Here, transnational youth’s funds of knowledge are understood through metonymies of relationality, circulation, multiplicity, and regeneration (see Figure 5). These metonymic shifts give rise to a new conceptual metaphor that frames bilingualism not as a bounded skill set but as a living network of practices that extend across time, space, and sociopolitical contexts (Serna-Gutiérrez, forthcoming).

Drawing from interdisciplinary work around fungi and mycelium, the metaphor TRANSNATIONAL YOUTH FUNDS OF KNOWLEDGE ARE MYCELIAL NETWORKS emphasizes entanglement, adaptation, and non-linearity. As Serna-Gutiérrez (forthcoming) notes, Sheldrake (2020) describes mycelium as operating through flexible, non-linear expansion, a form of growth that resists centralized control and instead unfolds through distributed, responsive pathways. This understanding aligns closely with how transnational youth develop and mobilize their linguistic repertoires across contexts shaped by migration, schooling, and racialization. Their languaging practices do not follow predictable or bounded trajectories, rather, they emerge through ongoing negotiation within overlapping social fields. In this sense, bilingualism as a resource is not a static possession but a dynamic process that reflects the adaptive, regenerative qualities of mycelial systems. The metaphor thus shifts the focus from linguistic competence as an individual attribute to linguistic practice as an emergent property of relational networks.

At the same time, Serna-Gutiérrez’s (forthcoming) engagement with Anna Tsing deepens this framing by foregrounding the role of “friction” in shaping global connections. Tsing (2005) reminds us that movement across space is never smooth. It is constituted through uneven encounters that produce both constraint and possibility. This insight is critical for understanding transnational youth’s experiences, as their languaging practices are formed within and against the raciolinguistic ideologies that circulate across borders. Extending this perspective, Serna-Gutiérrez (forthcoming) also draws on Ostendorf-Rodríguez (2023) to emphasize the situated and historically embedded nature of transnational knowledge production, underscoring how language practices are always tied to lived experiences of migration, community, and identity. Together, these theoretical contributions reinforce the argument advanced in this article: that reimagining bilingualism through a mycelial lens not only disrupts deficit-oriented narratives but also provides a more accurate account of the complex, adaptive, and relational nature of transnational youth’s funds of knowledge.

#### 4.1.2. Expanding Metaphorical Representations of Translanguaging

This framing also extends bilingualism as right beyond access to language instruction or recognition of linguistic diversity. It positions transnational youth’s ways of knowing, speaking, and being as inherently valuable and worthy of sustenance within educational systems. A mycelial perspective demands that schools move beyond additive or even pluralistic models of bilingualism and instead cultivate conditions where these networks can thrive. This includes recognizing translanguaging as an ontoepistemic practice that is relational, historically embedded, and capable of resisting and reworking raciolinguistic ideologies. Although metaphors for understanding translanguaging exist, we see the dynamic spread, growth, and resistant lived movement of mycelium as a more accurate cognitive representation of translanguaging, compared to the bridge metaphor (Ashari et al., 2025) that produces a false cognitive understanding of translanguaging as “bridging,” “switching between,” or “connecting” two distinct languages or the “flowing corriente (current)” metaphor (Adams Corral & Sayer, 2024) that acts to cognitively produce a “bounded by river banks,” “uni-directional flow” of languaging; of which translanguaging is not.

The TRANSNATIONAL YOUTH FUNDS OF KNOWLEDGE ARE MYCELIAL NETWORKS metaphor goes beyond just language use and foregrounds the importance of connection and interdependence in ways that challenge individualistic conceptions of language learning. Just as mycelial networks facilitate communication and resource-sharing across diverse organisms, transnational youth’s funds of knowledge are co-constructed through family, community, and transnational social fields. These networks carry with them histories of migration, cultural practices, and linguistic repertoires that cannot be contained within the boundaries (riverbanks) of named languages or nation-states. In this way, bilingualism as a resource becomes inseparable from broader questions of epistemic justice and decoloniality.

The TRANSNATIONAL YOUTH FUNDS OF KNOWLEDGE ARE MYCELIAL NETWORKS metaphor functions as both a conceptual and political intervention within the bilingualism as a resource and bilingualism as a right orientations. It shifts the discourse from remediation to recognition, from containment to connectivity, and from deficit to abundance. By transforming the underlying metonymic and metaphorical structures that shape how bilingualism is understood, this new metaphor opens possibilities for more just and equitable educational futures that honor the full complexity of transnational youth’s languaging lives as dynamic, interconnected, and profoundly generative. This new story provides the script for transnational youth to see themselves as the main characters, well prepared with their transnational funds of knowledge, to be successful in any new social and educational setting.

## 5. Concluding Thoughts and a Call to Action

It’s so easy to submit to pre-packaged and prefabricated sentences and stories, accepting them as accurate representations of one’s own consciousness and experience…but there are degrees of submission. Anyone who even partially resists the templates on offer is by my measure some kind of artist…The community that rips a Creole tongue, or a patois, from the pages of a colonizer’s textbook-they have made art-Small and peculiar acts of art-and therefore of resistance-are everywhere.

(Smith, 2025, p. 287)

### 5.1. Addressing and Remythifying a Common Myth About Schooling in the United States

Over 200 years into the experiment of public education in the United States (one of the most culturally and linguistically diverse countries in the world), access to and value of bilingualism for transnational youth, today, continues to be linked to a perceived and/or real social distance from white, English-speaking culture and communities.^6^ Golash-Boza sums this well, stating, “the implication is that although we purport to celebrate multiculturalism, it appears that there continue to be disadvantages associated with nonconformity to Anglo culture” (p. 750). Above, we laid out how this cultural and linguistic nonconformity is judged, first by others, then by transnational students, themselves, through rapid, cognitive simplifications of highlighted linguistic features (e.g., accent, difference in word choice, non-English, and/or holistic practices, such as translanguaging, that are deemed broken) that stand in place for a summarized/generalized metaphor of transnational youth’s bilingualism is linguistic homelessness and a societal/educational problem to solve. The importance is not whether or not people believe these simplifications; the importance is that they create a story of life that helps those in power and those subjected to lesser power understand their place in the world. “It is perhaps because we know these simplifications to be impossible that we insist upon them so passionately…they describe a people, by defining them against other people—but the people being described are ourselves” (Smith, 2025, p. 191).

**Figure 6:**
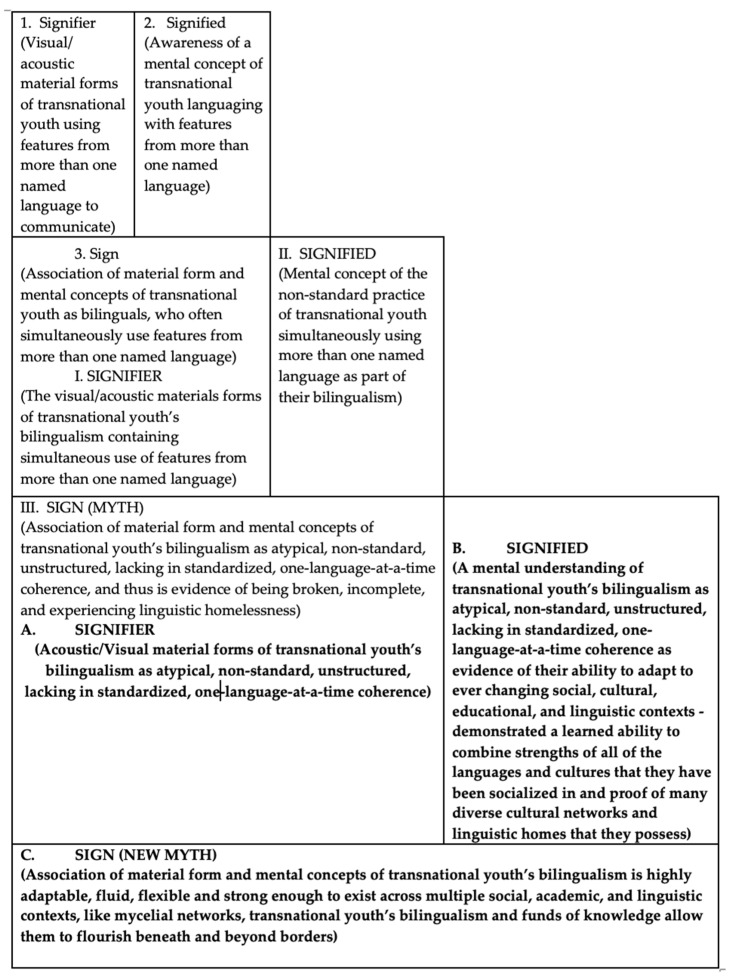
The three semiological chains of language, myth, and a new myth.

What Smith may be alluding to in the above quote is the social semiotic concept of mythologies—a kind of speech, a system of linguistic messages that attach themselves to any historical event or concept and, in doing so, have an agenda that promotes a certain way of thinking about the world, whether that way of thinking has been deeply considered/vetted or not (Barthes, 1957/1972). Mythologies (also closely aligned to “folk theory,” Przymus, 2025) are rarely questioned and are usually accepted as normalized knowledge. Mythologies simplify life and tell us (humans in this world) where we belong. Most myths are produced by those in power of disseminating information. Figure 6 illustrates how myths are a second-level system or semiological chain, built off of the first-level relationship (the closest to the truth) between a signifier (visual/acoustic forms of a concept) and a signified (mental awareness of the concept), resulting in a meaning-making “sign.” The second-level semiological chain is built off of the end (the sign) of the first-level system, converting the “sign” into a new “signifier,” starting a new chain. Figure 6 starts with what is considered “language,” or simply the existence of transnational youth producing visual/acoustic material forms that contain linguistic features from more than one named language (e.g., Spanish, English). When these material forms are heard or seen by others, they produce mental concepts in the interlocutors’ brains, resulting in a “signified” that these youth use more than one named language to communicate. Together, the association of material forms and mental concepts produces a meaningful sign of transnational youth as bilinguals, who often simultaneously use features from more than one named language. All myth needs are this existing “sign” and the motivation to build off of this to produce and introduce into society’s shared consciousness, a new way of thinking/talking about these youth’s bilingualism.

We see in Figure 6 that the existing sign from the first chain is picked up by myth producers who wish to focus on the fact that these youth’s bilingualism consists of “atypical” language use of simultaneously using features from multiple named languages (i.e., translanguaging) to produce a new “sign” or *myth* that transnational youth’s bilingualism is atypical, non-standard, unstructured, lacking in standardized, one-language-at-a-time coherence, and thus is evidence of being broken, incomplete, and experiencing linguistic homelessness.

#### Putting Forth a New Myth

Much discourse in society stops here, but it does not have to. The overall purpose of our paper is to convince readers that our language matters and influences thought. We too, academics and transnational youth, can also play in the myth-making game. With our new metaphor, above, that rejects the linguistic homelessness metaphor by demonstrating that transnational youth’s bilingualism actually is made up of a vast network (like mycelium) of cultural and linguistic structures, due to having lived in multiple contexts, we, too, have the potential to influence thought about these youth. This is the process of remythification or the practice of starting with the end sign of the second-level semiological chain (the myth) and using that myth as the beginning of a new signifier (Przymus, 2025). This new signifier uses the language of the myth, that transnational youth’s bilingualism is atypical, non-standard, unstructured, lacking in standardized, one-language-at-a-time coherence, but reframes this as a mental concept (signified) of evidence of a linguistic strength or the ability to adapt to ever-changing social, cultural, educational, and linguistic contexts, a learned ability to combine strengths of all of the languages and cultures that these youth have been socialized in, and proof of the many diverse cultural networks and linguistic homes that they possess. This, then, results in a new myth, language, and way of thinking/talking about these youth and their bilingualism as highly adaptable, fluid, flexible and strong enough to exist across multiple social, academic, and linguistic contexts, like mycelial networks, allowing them to flourish beneath and beyond borders—or in other words, viewing their bilingualism as a resource. Like mycelium, which acts as an ecosystem’s communication and resource-sharing system, this new language and new way of thinking/talking about transnational youth reorients their communication to its own resources-sharing ecosystem.

### 5.2. A Call to Action

By unearthing the root of the bilingualism-as-problem orientation for transnational youth, we can learn from this to reorient bilingualism from a problem to be solved to a resource to be harnessed and a right to be enjoyed for transnational youth. Since the problem orientation is rooted in cognitive metonymies and metaphors, we must also work to change those mental shortcuts and stories for understanding the world to different and more positive metonymies and metaphors. Here, we take our theoretical proposition for a new metaphor, TRANSNATIONAL YOUTH’S FUNDS OF KNOWLEDGE ARE MYCELIAL NETWORKS, and call for real, concrete pedagogies that support this metaphor. One such example that leverages the real, transnational networks created by transnational youth is the methodology and practice of epistolary diasporic saberes (knowledges), or the opening-up of knowledge production and demonstration via tapping into a long-standing, transnational tradition of letter writing as a way of nurturing transnational networks and connections between the lands/spaces that transnational youth inhabit. The recirculation of transnational knowledge, memories, translingual practices, etc. present in epistolary practices provide for language practice and is a concrete valuation of these students’ past, present, and future identities.

The production and continued use of a new metaphor that visually produces vivid cognitive representations and stories of transnational youth’s bilingualism as vast connected networks of linguistic structures, that transcend boundaries, that spread from context to context, and that are resilient in the face of new contexts, has the power and potential to reframe these youth’s bilingualism from something lacking and as a problem to be solved to something rich and expansive, as a resource for social and educational success. And although the scope of this current paper does not allow space to deeply discuss specific educational implications that could arise from such a change in language, it is not hard to dream of a different future for transnational youth. In classrooms, teachers could recognize transnational students’ vast linguistic and cultural resources to teach and assess them better. For educational decision-makers, curriculum, language, and assessment policies could be reenvisioned to think beyond the monolingual paradigm that leaves transnational students on the margins (Przymus, 2016). Finally, for researchers (the vast audience reading this), we must constantly be open to new theories that continue to further the knowledge of who transnational youth are, work with transnational youth as co-creators of knowledge, and together strive to give up bad language, for good.

## Data Availability

The original contributions presented in this study are included in the article. Further inquiries can be directed to the corresponding author.

## Notes

1 Across this special issue, the term transnational youth is used as an overall term for youth “who live across two or more nations and who attend school in one or more of the nations they call home” (Skerrett, 2020, p. 499; Przymus et al., 2022).

2 Although an adjective for much of the Western hemisphere, American is metonymically understood to stand in place for just the United States of America, the context of this article (Przymus, 2025).

3 It should be noted that in the U.S. context, white, first-language English-speaking students are often glorified and celebrated for their emergent bilingualism that can result from dual-language, bilingual education or foreign language instruction, even when their proficiency levels in the language other than English often are far below that of their transnational student peers’ proficiency levels in English (Schwartz, 2023; Valdés, 1997).

4 Gros quotes Rostand’s (1990) play, *L’Aiglon*, as a way of demonstrating the history and the extent of how language has been used to cast shame upon others.

5 A grammatically incorrect Spanish phrase, meaning “I don’t know,” meant to draw derogatory attention to the lack of standard Spanish language fluency and highlight transnational youth’s different ways of Speaking and being.

6 Although the focus of this article is the U.S. context, we believe that the reproduction of social and educational inequities exist all around the globe, where transnational students live and attend school, and that raciolinguistic ideologies linked to “English-language curricula and Eurocentric standards” are also at the social cognitive foundation of these inequities (Day & Zhang, 2026, p. 42; see this cited article for a discussion of the use of AI as a means of resisting these educational and social inequalities).
